# Nonfatal Injuries Sustained in Mass Shootings in the US, 2012–2019: Injury Diagnosis Matrix, Incident Context, and Public Health Considerations

**DOI:** 10.5811/westjem.58395

**Published:** 2023-05-02

**Authors:** Matthew P. Czaja, Chadd K. Kraus, Su Phyo, Patrick Olivieri, Dalier R. Mederos, Ivan Puente, Salman Mohammed, Ross P. Berkeley, David Slattery, Thomas H. Gildea, Claire Hardman, Brandi Palmer, Melissa L. Whitmill, Una Aluyen, Jeffery M Pinnow, Amanda Young, Carly D. Eastin, Nurani M. Kester, Kaitlyn R. Works, Andrew N. Pfeffer, Aleksander W. Keller, Adam Tobias, Benjamin Li, Brian Yorkgitis, Soheil Saadat, Mark I. Langdorf

**Affiliations:** * Ponce Health Sciences University School of Medicine, Ponce, Puerto Rico; † Geisinger Emergency Medicine, Danville, Pennsylvania; ‡ Touro University Nevada College of Osteopathic Medicine, Henderson, Nevada; § Valley Health Emergency Medicine, Las Vegas, Nevada; || Broward Health Medical Center, Division of Trauma and Critical Care Services, Fort Lauderdale, Florida; # Kirk Kerkorian School of Medicine at UNLV, Department of Emergency Medicine, Las Vegas, Nevada; ¶ St. Louise Regional Hospital, Department of Emergency Medicine, Gilroy, California; ** Santa Clara Valley Medical Center, Department of Emergency Medicine, San Jose, California; †† Wright State University Boonshoft School of Medicine, Department of Surgery, Dayton, Ohio; ‡‡ Kettering Health Main Campus, Trauma Research Program, Kettering, Ohio; * Kettering Health Main Campus, Division of Acute Care Surgery, Critical Care, and Trauma, Department of Surgery, Kettering, Ohio; † Texas Tech University Health Sciences Center School of Medicine, Department of Emergency Medicine, Odessa, Texas; ‡ Medical Center Hospital, Department of Emergency Medicine, Odessa, Texas; § University of Arkansas for Medical Sciences, Department of Emergency Medicine, Little Rock, Arkansas; || University of Texas Health Science Center at San Antonio, Department of Emergency Medicine, San Antonio, Texas; # Vanderbilt University Medical Center, Department of Emergency Medicine, Nashville, Tennessee; ¶ University of Pittsburgh Medical Center, Department of Emergency Medicine, Pittsburgh, Pennsylvania; ** Denver Health, Department of Emergency Medicine, Denver, Colorado; †† University of Florida College of Medicine, Division of Acute Care Surgery, Department of Surgery, Jacksonville, Florida; ‡‡ University of California Irvine School of Medicine, Department of Emergency Medicine, Irvine, California

## Abstract

**Introduction:**

The epidemic of gun violence in the United States (US) is exacerbated by frequent mass shootings. In 2021, there were 698 mass shootings in the US, resulting in 705 deaths and 2,830 injuries. This is a companion paper to a publication in JAMA Network Open, in which the nonfatal outcomes of victims of mass shootings have been only partially described.

**Methods:**

We gathered clinical and logistic information from 31 hospitals in the US about 403 survivors of 13 mass shootings, each event involving greater than 10 injuries, from 2012–19. Local champions in emergency medicine and trauma surgery provided clinical data from electronic health records within 24 hours of a mass shooting. We organized descriptive statistics of individual-level diagnoses recorded in medical records using International Classification of Diseases codes, according to the Barell Injury Diagnosis Matrix (BIDM), a standardized tool that classifies 12 types of injuries within 36 body regions.

**Results:**

Of the 403 patients who were evaluated at a hospital, 364 sustained physical injuries—252 by gunshot wound (GSW) and 112 by non-ballistic trauma—and 39 were uninjured. Fifty patients had 75 psychiatric diagnoses. Nearly 10% of victims came to the hospital for symptoms triggered by, but not directly related to, the shooting, or for exacerbations of underlying conditions. There were 362 gunshot wounds recorded in the Barell Matrix (1.44 per patient). The Emergency Severity Index (ESI) distribution was skewed toward higher acuity than typical for an emergency department (ED), with 15.1% ESI 1 and 17.6% ESI 2 patients. Semi-automatic firearms were used in 100% of these civilian public mass shootings, with 50 total weapons for 13 shootings (Route 91 Harvest Festival, Las Vegas. 24). Assailant motivations were reported to be associated with hate crimes in 23.1%.

**Conclusion:**

Survivors of mass shootings have substantial morbidity and characteristic injury distribution, but 37% of victims had no GSW. Law enforcement, emergency medical systems, and hospital and ED disaster planners can use this information for injury mitigation and public policy planning. The BIDM is useful to organize data regarding gun violence injuries. We call for additional research funding to prevent and mitigate interpersonal firearm injuries, and for the National Violent Death Reporting System to expand tracking of injuries, their sequelae, complications, and societal costs.

## INTRODUCTION

Civilian public mass shootings (CPMS) are increasing in frequency and are the leading cause of potential years of lost life in the United States (US).^1^ Nonfatal interpersonal firearm injuries outnumber deaths two- to threefold.^2^ As greater than 75% of all firearm deaths occur prior to hospital arrival, reports that focus on mass shooting deaths provide an incomplete picture of the medical resources required to care for injured victims and provide inadequate information for effective hospital and emergency department (ED) disaster planning.^2^ While most research on firearm-related injuries, including reports on mass shootings, focus on deaths, less is known about injury patterns and outcomes among survivors, including those injured by non-ballistic means.

Mass shootings are a complex subset of the larger firearm violence epidemic in the US. Some are random, but others are associated with hate crime ideology or a response to bullying or social isolation.^3^ One factor common to CPMS is the use of automatic or semi-automatic firearms (SAF).^3^ “Assault rifles,” generally defined as selective-fire rifles that use intermediate power ammunition fed from a detachable magazine (often high capacity), cause greater mortality and morbidity in mass shootings than non-automatic weapons.^4^ The kinetic firepower and resulting damage of these SAFs is potentially orders of magnitude greater than that of a musket ball used in the late 18^th^ century, at the time the Second Amendment was adopted, and is further compounded by the increased rate of fire of modern weapons.^5^,^6^

This is a companion manuscript to the *JAMA Network Open* paper entitled “Injury Characteristics, Outcomes, and Health Care Services Use Associated with Nonfatal Injuries Sustained in Mass Shootings in the US, 2012–2019.”^7^ Our report provides a greater level of detail on the injury epidemiology of the 13 mass shootings previously analyzed, by organizing all traumatic diagnoses according to the Barell Injury Diagnosis Matrix (BIDM).^8^ We also present atraumatic diagnoses and illnesses, including sequelae of trauma. Lastly, this report addresses mass shooting settings, firearm type and legality, and hate crime associations, with expanded discussion of the research processes and limitations.

## METHODS

This retrospective case series of 403 patients reports 13 CPMSs with greater than 10 injuries per event from 2012–19. The study design and data abstraction methods have been reported previously.^7^ Briefly, we identified these CPMS incidents via public databases, The Violence Project (TVP),^9^ and *Mother Jones*,^10^ and contacted local champions to report data from 31 hospitals that received injured victims to report data to a central hub. The study was deemed exempt from institutional review board (IRB) approval at the central site. Data were abstracted from primary medical records of victims presenting within 24 hours after the CPMS, and IRB approval was obtained at each spoke center.

**Population Health Research Capsule**

What do we already know about this issue?

*The firearm violence epidemic in the US is exacerbated by increasingly frequent mass shooting, involving significant deaths and a greater number of non-fatal injuries*.

What was the research question?

*We describe the morbidity (gunshot wounds and other) among mass shooting survivors and discuss the types of firearms used and public health implications*.

What was the major finding of the study?

*In 13 mass shootings, 887 nonfatal injuries were associated with semi-automatic firearm use. There were 2.88 GSW injuries, and 1.56 non-GSW injuries per patient*.

How does this improve population health?

*Law enforcement, EMS, and hospital disaster committees may use these insights into mass shooting morbidity for injury mitigation and public policy planning*

We used best-practice methods of retrospective chart review.^11^ We excluded deaths at the scene, in the emergency department (ED), and in the operating room during initial surgery. We included all patients from the CPMS, including those not injured by GSW, as well as uninjured patients presenting for medical complaints. To add context to the injured victims, we summarize the incident-level data retrieved from *TVP* database on type, number and legality of firearms used, hate crime components, and reported motive.^9^ We collected Emergency Severity Index (ESI) triage levels on 232 of 403 patient (57.6%). For the other 171 victims, we assigned an ESI based on diagnosis, injury type, and projected resources used as per the definition for each ESI level.^12^

We compiled patient-level data on ED and inpatient diagnoses from medical records according to the *International Classification of Diseases*, Ninth Revision, Clinical Modification (ICD-9-CM) and Tenth Revision (ICD-10-CM) codes.^13^,^14^ We employed the BIDM, a standardized epidemiological tool that presents ICD-9-CM codes describing trauma in a two-dimensional array (matrix) of 36 body region rows and 12 nature-of-injury columns.^8^ To deal with the ICD-10-CM codes in our sample, we used an online converter tool to translate these codes into their ICD-9-CM equivalents.^15^ For an additional layer of precision, we also referenced the Injury Mortality Diagnosis Matrix, which is a similar matrix using ICD-10-CM codes.^16^ We chose to model this study’s CPMS injury matrix on the BIDM given its widespread application on morbidity data, as opposed to mortality/cause-of-death data.^17^

We made several modifications to the BIDM to more appropriately present CPMS-specific traumatic diagnosis codes. In this study, the mass shooting injury matrix (MSIM) has an additional nature-of-injury column, “Gunshot Wound,” to describe penetrating open wounds caused explicitly by GSWs. Therefore, non-GSW penetrating open wounds, lacerations, and abrasions are described in the column “Laceration and Abrasion.” Such a distinction is not possible in the unmodified BIDM. The BIDM also features three types of traumatic brain injuries (TBI): “Type 1” describes intracranial, and “Type 2” and “Type 3” describe extracranial trauma, with the latter distinguishable only by loss-of-consciousness status. The MSIM features only two types of TBI, “Intracranial” and “Extracranial.” Next, we removed “Trunk” and “Burns” because our dataset did not contain any of these codes (ie, unspecified thorax trauma). Finally, we also removed “System-wide and Late Effects (Row 36),” as we reported these diagnosis codes separately from the MSIM.

For purposes of categorizing firearms used in CPMS in this report, we defined a SAF, whether pistol or rifle, as one that places the subsequent round in the chamber and then requires the user to depress the trigger again to fire the next round.^18^ Non-SAFs require additional actions by the user to fire the next round, other than pulling the trigger.^18^ The term “assault weapon” generally refers to a SAF with a detachable, large-capacity magazine and additional components that may include a pistol grip, a forward grip, and/or a flash suppressor.^19^ We relied on descriptions of the weapons used in mass shootings by *TVP*^9^ and did not independently verify the types of weapons used.

## RESULTS

This study describes 13 CPMSs from 2012–2019 across nine US states (Table 1). Three of the mass shootings occurred at religious sites, three at bars/nightclubs, and two each at schools and concerts/festivals. All shootings featured SAFs: 9 of 13 (69.2%) involved at least one semi-automatic assault rifle (SAAR), and 4 of 13 (30.8%) only involved semi-automatic pistols (SAP). A total of 50 firearms (3.85 per CPMS) were used by the perpetrators. Excluding the Las Vegas CPMS, which featured 24 firearms, there were 26 firearms used in the other 12 incidents (2.17 per shooting). There were 30 SAARs, 13 SAPs, three shotguns, three other handguns, and one bolt-action (non-automatic) rifle.

According to available public data, at least 32 of 50 (64%) firearms were obtained legally for six mass shootings. Only three firearms used in one CPMS were known to have been obtained illegally. Most legally obtained firearms were purchased from a federal licensed dealer, including all 24 firearms used in the Las Vegas CPMS. One legal firearm was bought in a private sale.

**Figure 1:**
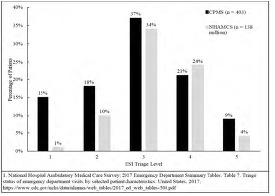
Distribution of Emergency Severity Index triage level at the primary receiving hospital for 403 survivors of 13 civilian public mass shootings in the United States (2012–19) compared to data from the 2017 National Hospital Ambulatory Medical Care Survey.

**Figure 2:**
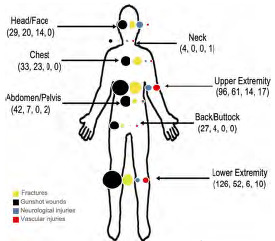
Anatomic distribution of nonfatal gunshot wounds and other trauma sustained by 403 survivors of 13 civilian public mass shootings in the United States (2012–19). Colored circles are proportional to the number of coded injuries, with black denoting gunshot wounds (n=357), yellow, fracture (n=157), blue, neurologic (n=34), and red, vascular (n=30).

Figure 1 shows that the distribution of ESI categories in this study’s 403 mass shooting survivors from disaster situations was skewed to the right, representing substantially higher acuity when compared to a national US sample of 138 million patients in 2017 from the National Center of Health Statistics.^20^ Figure 2 shows the anatomic distribution of four trauma subtypes: GSW; fracture; neurologic; and vascular trauma, with colored-dot sizes proportional to frequency of injury at each anatomic location.

### Mass Shooting Injury Diagnosis Matrix

There were 897 traumatic diagnoses recorded in the MSIM (Table 2) in total, equating to 2.48 per injured patient (364). Of these diagnoses, 725 (80%) were caused by GSW-related trauma and 172 (20%) were from other blunt trauma (eg, fall, stampede, trampling). There were almost twice as many traumatic diagnoses per GSW patient than for non-GSW mass shooting victims, reflecting the complicated nature of these injuries. The 725 GSW-related diagnoses for 252 GSW patients equates to 2.88/patient vs 172 non-GSW diagnoses for 112 patients, or 1.56/patient. For GSW victims, the most common forms of trauma involved fractures (163) and internal organ injuries (113). For non-GSW victims, lacerations/abrasions (60), soft tissue contusions (55), and musculoskeletal strains (33) accounted for most injuries. For all patients, the most frequent anatomic regions of trauma involved the chest/thorax (113), followed by the abdomen (89), and shoulder/upper arm/axilla (89).

### Internal Organs

The most common internal injuries from GSWs were 48 abdominal/retroperitoneal (including kidney), 41 thoracic, 13 intracranial, and 11 urogenital/pelvis. In addition, there were four myocardial infarctions, two injuries from blunt trauma, and two from pre-existing coronary artery disease. These injuries resulted in 64 diagnoses of organ failure and shock: 30 acute blood loss anemia; 11 hemorrhagic shock; 9 acute respiratory failure; and 14 other various organ failure diagnoses.

### Musculoskeletal

There were 196 total musculoskeletal diagnoses. The most common were wrist/hand (30), ribs (23), lower leg and ankle (18), upper extremity (17), foot (14), face (12), and skull (8). There were three reported cases of compartment syndrome of the leg associated with GSW trauma.

### Neurologic

There were 44 patients with 51 neurologic trauma diagnoses. Thirty-one of these patients (70.4%) had GSW, and 13 diagnoses were related to blunt head trauma (concussions). For GSW victims, there were 24 with peripheral nerve injuries and six patients with 13 intracranial TBIs (eg, epidural, subdural, subarachnoid, brain parenchymal). One patient had a traumatic spinal cord injury (not recorded in MSIM). The most common peripheral nerve injuries involved the lower extremity (of eight peripheral nerve injuries, four were to sciatic nerves and four were other leg nerve injuries), and the upper arm/axilla and forearm/elbow/wrist (seven each).

### Vascular

Vascular injuries were most commonly paired with peripheral nerve injuries (22 with nerve injuries and 12 isolated). There were 34 patients with 34 vascular injuries, comprised of 17 upper extremity, 12 lower extremity, two abdominal, and one neck (two unknown).

### Complications/Sequelae

There were 88 diagnoses related to complications and sequelae of trauma: 43 involving foreign bodies; 11 gastrointestinal with ileus/constipation (opioid-induced and other); six with venous thromboembolism; two with ostomies; and two with wound dehiscence during the index hospitalization.

### Infectious/Metabolic

There were 20 reported infectious diagnoses among 144 admitted patients (13.9% of all victims) and 37 diagnoses involving metabolic derangements, most frequently hypokalemia (11).

### Atraumatic

Thirty-nine patients did not sustain any physical injury, and 21 of these cases (53.8%) involved psychiatric diagnoses. The others had a combination of acute concerns associated with the shooting (ie, syncope, hearing loss from gunfire), exacerbations of pre-existing chronic conditions, and occupational exposure to blood products (first responders). Some injured patients concurrently had non-traumatic diagnoses, especially admitted patients. Four patients had asthma exacerbations, four had hearing loss, three had cardiovascular disease (two atraumatic myocardial infarctions and one hypertensive emergency), and three had obstetric concerns.

### Psychiatric

Overall, 50 patients had 75 psychiatric diagnoses (1.50 per patient); Twenty-nine of these 50 patients (58.0%) also had physical trauma, and 21 (42.0%) did not have a physical injury. Thirty-five (46.7%) of the psychiatric diagnoses were anxiety/panic/adjustment disorders. Fifteen patients (20%) were diagnosed with acute stress disorder (ASD), seven (9.3%) with major depressive disorder/depressive symptoms, and six (8.0%) with post-traumatic stress disorder (PTSD), indicating a hospital stay longer than 30 days according to the *Diagnostic and Statistical Manual of Mental Disorders*, Fifth Edition.^21^

## DISCUSSION

This report focuses on injuries and related CPMS conditions, rather than deaths, to broaden and further describe the morbidity of victims, along with the societal and healthcare sequelae. This study is an additional analysis of a prior publication, in which we now report further detail on mass shooting settings, firearm type and legality, reported hate crime association, non-GSW trauma and illnesses, and research processes and limitations.^7^

As per Table 1, mass shootings occur in a variety of settings, including concerts, schools, places of worship, social gathering sites like bars, military bases, hospitals, and workplaces.^22^ Over 90% occur within one mile of places frequented by children (eg, school, park).^23^ Figure 1 demonstrates that CPMSs survivors skew toward substantially higher acuity for triage severity and anticipated care resources compared with a national comprehensive sample of EDs in the US.^20^

Prehospital planning and mass casualty incident training simulations are key to preventing loss of life, especially given the shift toward higher acuity. There is ample evidence suggesting that prehospital training programs and tourniquet training for laypersons increase survival.^25^,^26^,^27^ Some public gathering places now have “STOP THE BLEED” kits, analogous to previous deployment of cardiac defibrillators. For example, in 2022 the city of Chicago deployed 550 STOP THE BLEED kits in 350 locations throughout the city.^28^

Brown and Goodin^29^ reported that 44% of fatalities and 62% of all CPMS victims were associated with SAAR use. In these 13 CPMSs, there were 157 deaths and 887 nonfatal injuries, all of which were associated with SAF use.^7^ Nine incidents involved SAAR use, corresponding to 147 of 157 (94%) deaths and 810 of 887 (91%) nonfatal injuries, although approximately one-third of these were non-GSW trauma. The ability to fire many bullets from a high-capacity magazine of a SAF is a direct cause of multiple injuries and deaths, and their lethality far exceeds anything likely imagined by the authors of the Second Amendment.^5^ Public policy recommendations include restriction on purchase of high-capacity magazines as an important plank in mitigating potential injuries and deaths of mass shootings.^30^

### Policy Implications

High-capacity SAF (which include assault rifles) are used in 20–58% of all firearm mass murders, but are used in a particularly high proportion of public mass shootings.^3^,^4^,^29^ Civilian public mass shootings with SAFs result in substantially more fatal and especially non-fatal victims due to their rapid-fire abilities, enabling the perpetrator to indiscriminately target victims in enclosed spaces or large gatherings.^3^,^4^,^29^ The 1994 federal ban on assault weapons and large-capacity (>10 rounds) ammunition magazines had exemptions and loopholes that limited its short-term effects, but its expiration in 2004 was followed by an increase in the use of these weapons in mass shootings and other crimes.^31^,^32^ Data suggests that policy measures involving state-level restrictions on large-capacity magazines may reduce mass shootings.^4^

Evidence from 130 studies in 10 countries demonstrates that, in other nations, the simultaneous implementation of laws targeting multiple firearms restrictions is associated with reductions in firearm deaths.^33^ Laws restricting the purchase of (eg, background checks) and access to firearms (eg, safer storage) are also associated with lower rates of intimate partner homicides and unintentional firearms deaths in children, respectively.^33^ Furthermore, laws requiring permits to purchase a gun are also associated with a lower incidence of mass public shootings, and bans on large-capacity magazines are associated with fewer fatalities and nonfatal injuries when such events do occur.^34^

Our findings from mass shooting events represent a subset of US national firearm injuries and deaths. The US Centers for Disease Control and Prevention established the National Violent Death Reporting System (NVDRS) in 2002, with six states reporting.^35^,^36^ Currently all 50 states, the District of Columbia, and Puerto Rico report their data. This robust dataset tracks all types of firearm deaths, including intentional, unintentional, those from interpersonal violence, legal intervention, and undetermined intent. However, the NVDRS does not track *nonfatal* firearm injuries as described here.^36^ Research funding to study important aspects of firearm death is now available and has been distributed to 16 projects to date. However, none of these currently funded projects focus on mass shooting intervention or prevention. Ten state government agencies have received funding to enhance death surveillance and reporting, again excluding firearm injuries.

The NVDRS applies the principles of public health research and intervention pioneered by Dr. William Haddon, the first director of the National Highway Safety Administration, whose use of the scientific approach led to dramatically reduced morbidity and mortality of highway crashes over decades. Application of these principles has promise to similarly reduce both injuries and deaths from firearm violence.^37^

### Anatomic and Organ System Injuries in the Barell Injury Diagnosis Matrix

The BIDM is a reliable and useful format for describing trauma-related morbidity. There is already a NVDRS, and this matrix could form the backbone of an analogous national violent *injury* reporting system.^38^ In this study, we modified the BIDM to distinguish between GSW and non-GSW open wounds and penetrating trauma. The ICD system is imperfect, as it was designed for billing purposes rather than clinical research. However, its use here as a clinical surrogate is widespread and relatively straightforward. We call for greater resources and funding to better capture CPMS data in trauma registries and to separate these data from other firearm violence. We also recognize the need for a universal definition for mass shooting for clinicians and public health workers. Finally, there is a need to separately track the GSW vs non-GSW injuries. Although we found most injuries were ballistic-related, 19.1% of the injuries were not.

### Mass Shootings Patient Conditions Apart from Trauma

While we did not collect long-term follow-up data, it is well known that victims of firearm injury and mass shootings suffer from higher rates of psychological illness.^39^ Children and adolescents may be especially vulnerable, suffering from higher rates of post-traumatic stress, suicide, depression, substance abuse, and anxiety.^40^ Hospitals should incorporate aftermath services that address the psychological sequelae of a CPMS into their emergency medical systems (EMS) disaster plans.

Psychiatric conditions were common among our patients from mass shootings. We found 12.4% of the victims presented for acute mental health issues; given the chaos of these incidents, the true proportion and impact on mental health is certainly higher. Fifteen patients were formally diagnosed with ASD, and six were diagnosed with PTSD by the time of hospital discharge. These 21 patients formed almost one-third (28%) of all psychiatric diagnoses. As a key difference between ASD and PTSD involves the duration of symptoms (3–30 days vs > 30 days),^21^ it is plausible that some of the patients with ASD may have subsequently developed PTSD.

Furthermore, the incidence of psychiatric sequelae in our sample is likely under-reported, as some patients never presented with acute psychological distress, and 256 of all 403 patients (63.5%) were discharged from the ED before any detailed evaluation of their emotional state. Therefore, the incidence of 5.2% (21/403) should be considered a minimum proportion. In the acute post-disaster period, one study found 20% of men and 36% of women met criteria for PTSD, the most prevalent psychiatric disorder. One-half of women and one-fourth of men with PTSD also met criteria for other psychiatric diagnoses, most commonly major depression.^40^

Survivors of GSWs may experience negative psychiatric outcomes for years after.^41^ The diagnoses not included in the MSIM speak to this point. We found 39 patients with only non-traumatic diagnoses (9.7%) and an additional 50 patients (12.4%) with 75 more psychiatric diagnoses, for a total of 22.1% with only or additional non-injury diagnoses. It is unlikely that all potential psychiatric diagnoses were contemporaneously captured due to the chaos and short evaluation time of many patients; therefore, this report likely underestimates the true number. Vela et al^41^ evaluated GSW victims (not specifically from CPMS) and found that combined alcohol and substance use increased from 30.8% pre-to 44.0% post-GSW. Subjects up to five years after GSW had lower than comparison population scores on Global Physical Health (45 [11]; *P* < .001), Global Mental Health (48 [11]; *P* = .03), and Physical Function (45 [12]; *P* < .001) on the National Institutes of Health’s Patient-Reported Outcome Measure Information System.^42^ Furthermore, they found 48.6% of their subjects screened positive for probable PTSD, far greater than the 12.4% found here.

Physical problems for victims of a CPMS last far beyond the acute care/initial hospitalization phase. Although we gathered systematic data on all injured patients during the index ED visit and subsequent hospitalization, and hospital charges for the following week, we recognize that healthcare costs and disability continue. An example from the dataset includes one patient from a CPMS who was shot in the extremity and presented for initial care the next day to his home hospital hundreds of miles away, with a complex long-bone fracture. His ongoing care included five major surgeries and follow-up visits for 2.5 years. until ultimately lost to follow-up. He accumulated $450,000 in medical charges, and, at the last documented visit, continued to suffer residual disability with work restrictions involving light duty only. Victims of mass shootings have complex and ongoing care needs. Therefore, this report should be considered an accurate description of only the initial phase of injury care. Further work is needed to better understand the comprehensive consequences of physical and psychological injury.

Communities, individuals, and healthcare workers who fall victim to CPMS can benefit from mental health resources such as critical incident stress debriefing (CISD).^43^,^44^ Per the US Department of Labor, CISD is a facilitator-led group process conducted soon after a traumatic event with individuals considered to be under stress from trauma exposure. In addition, psychosocial interventions, such as Psychological First Aid, Skills for Psychological Recovery, and Listen, Protect, and Connect: Psychological First Aid for Children and Parents, have been developed to aid victims.^45^,^46^ These programs should be incorporated into hospital- and EMS-level disaster plans to help individuals cope with the aftermath of CPMS.

### Emergency Planning

Our results also provide information for emergency planning and resource allocation preparation by community EDs in the event of a mass shooting. Any community ED could face, and should be prepared for, a mass casualty event from a civilian mass shooting. Injured victims could quickly overwhelm the resources of community hospitals that lack the advanced resources of a tertiary trauma or regional referral center. (Community hospitals received 194/403 [48.1%] of the patients in the current study.) Although 14/403 (3.5%) patients were ultimately transferred to centers with advanced resources, the initial stabilization and much of the comprehensive care were provided in non-trauma centers. Therefore, it is critical that these facilities prepare for the types and frequencies of injuries described here.

### Future Research

Important foci of research and public policy change include assessment of the potential impact of “smart guns,” which can only be fired by the registered user; increased background checks and waiting periods (including closing so-called “gun-show loopholes” that avoid background checks); appropriate application of concealed weapon permits; removal of tort liability protections for gun manufacturers; restriction of semi-automatic and automatic weapons; and restriction of large-capacity magazines, which is specifically important to mitigate the harm from CPMS described here.

## LIMITATIONS

### Neighborhood Shootings/Public Databases

This study focuses on mass shootings with >10 injuries, but numerous shootings occur daily (698 in the US in 2021) with multiple victims sustaining considerable morbidity along with death. These daily shootings do not fit within the Congressional Research Service definition of mass shootings, which excludes gang violence and shootings involving criminal profit.^47^ Furthermore, public databases have varying definitions of mass shootings.^48^ While the definition used here was purposely narrow to identify a large number of victims at sites with high potential for engaged local champions (see Site Recruitment, below), the injuries from neighborhood gang violence and criminal profit are no less devastating. In fact, these neighborhood shootings have been shown to garner less public attention from the media, another indicator of health and safety inequities in minority communities.^49^ The injury patterns, outcomes, and resource use reported here are likely generalizable to the larger firearm violence epidemic, with the caveat that neighborhood shootings may be less likely to involve SAFs with high-capacity magazines.

### Site Recruitment

Mass shooting site recruitment for this study required local champions at hospitals that treated victims from CPMSs. Some institutions were unwilling to contribute data to the study for fear of public relations damage (personal communication). In addition, some patients presented to non-teaching hospitals, which lacked either research infrastructure or interest to participate. Data on 377 patients (45% of our potential sample) from the Las Vegas Route 91 Harvest Festival CPMS were unobtainable due to site-related limitations.^7^ This reinforces our call for a national database of mass shooting deaths *and injuries*, not dependent on local cooperation.

### Data Collection

The true number of patients who presented to EDs for 12 of the CPMSs reported here are generally lower than publicly reported databases. By contrast, for the Las Vegas CPMS, local treating physicians reported that many patients were never registered or had incomplete documentation, given the volume, pace of influx, acuity, and arrival without identification.^50^ Chaos and communication breakdowns are common to all mass shootings, with one study finding that 13 of 17 (76.5%) incidents experienced a communication failure in the aftermath.^51^ Public databases are based on lay media and have different definitions; thus, the challenges we encountered highlight the importance of accurate information. Data collection was also hindered by age of records, legacy medical records, and IRB-specific restrictions, such as the exclusion of children, pregnant women, and police officers at some sites. Despite the difficulties in site recruitment and discrepancies with reported statistics, the 403 victims described in this study represent real patients, as opposed to media estimates.

## CONCLUSION

Mass shootings are common in the United States. In addition to further research on the human toll of these events, we call for additional study of the psychology of the perpetrators, the forensics of their weapons, abortive/prevention strategies, and the long-term physical and emotional impact on survivors. We advocate for the addition of firearm-related injuries to the existing infrastructure of the National Violent Death Reporting System. Only with proper research and funding will we best inform public policy to mitigate the enormous consequences of mass shootings.

## Figures and Tables

**Table 1:** Incident demographics of the 13 civilian public mass shootings in the United States from 2012–19.

CPMS Name	Date	Setting			Firearms used^b^			Hate crime association^c^	
		
		Location	Category^a^	Description	Quantity	Description	Legally purchased?	Prejudice(s)	Motive(s)
Midland-Odessa	08/31/2019	Midland and Odessa, TX	Other	Interstate 20	1	One SAAR	Yes, Private sale	No	No
Dayton	08/04/2019	Dayton, OH	Bar/Nightclub	Ned Peppers Bar	2	One SAAR (AR-15), one shotgun	Yes (All), Federally licensed dealer	Misogyny	No
Gilroy Garlic Festival	07/28/2019	Gilroy, CA	Concert/Festival	Gilroy Garlic Festival	1	One SAAR (AK-47)	Unavailable	Unavailable	Unavailable
Pittsburgh Synagogue	10/27/2018	Pittsburgh, PA	Religious locale	Tree of Life – Or L’Simcha Congregation	5	Three SAPs (Glock .357), one SAAR (AR-15), one shotgun	Unavailable	Racism (Jewish), Religious Hatred, Misogyny	Targeting racial/ethnic group, Anti-Semitism
Jacksonville Landing	08/26/2018	Jacksonville, FL	Bar/Nightclub	Good Luck Have Fun Game Bar, Videogame tournament	2	Two SAPs (.45-caliber & 9mm)	Unavailable	Unavailable	Unavailable
Marjory Stoneman Douglas High School	02/14/2018	Parkland, FL	School	Marjory Stoneman Douglas High School	1	One SAAR (M&P15)	Yes, Federally licensed dealer	Racism, Religious Hatred, Homophobia	Unavailable
Marshall County High School	01/18/2018	Benton, KY	School	Marshall County High School	1	One SAP (.22-caliber)	Unavailable	Unavailable	Unavailable
Sutherland Springs Church	11/05/2017	Sutherland Springs, TX	Religious locale	First Baptist Church	3	Two SAPs 22-caliber & 9mm), one SAAR (AR-556)	No (All), Unlawful purchas	No	No
Las Vegas	10/01/2017	Las Vegas, NV	Concert/Festival	Route 91 Harvest Music Festival	24	22 SAARs (AR-15 & AK-47), one bolt-action rifle, one revolver handgun	Yes (All), Federally licensed dealer	No	No
Burnette Chapel	09/24/2017	Antioch, TN	Religious locale	Burnette Chapel Church of Christ	2	Two SAPs (.40-caliber & 9mm)	Unavailable	Unavailable	Unavailable
Little Rock Nightclub	07/01/2017	Little Rock, AR	Bar/Nightclub	Power Ultra Lounge nightclub	3	Two handguns, one SAAR (AK-47)	Unavailable	Unavailable	Unavailable
Fort Lauderdale Airport	01/06/2017	Fort Lauderdale, FL	Other	Fort Lauderdale-Hollywood International Airport	1	One SAP (9mm)	Unavailable	No	No
Aurora Theater	07/20/2012	Aurora, CO	Other	Century 16 movie theater, Midnight screening of Dark Night Rises	4	Two SAPs (.40-caliber), one SAAR (M&P15), one 12-gauge shotgun	Yes (All), Federally licensed dealer	No	No
13 CPMS Incidents	2012–2019	9 US States	3 religious locales, 3 bars/nightclubs, 3 other settings, 2 schools, 2 concerts/festivals			30 SAARs, 13 SAPs, 3 shotguns, 3 other handguns, 1 other rifle	32 (64%) legal firearms	3 CPMS associated with prejudice	1 CPMS associated with antisemitic motive

a Setting categories and descriptions are based on information from Mother Jones (MJ) and The Violence Project (TVP), public databases, and lay press sources/public domain.

b All firearms used by the perpetrator(s) including those used to target victims and those found on scene even if not discharged. Information abstracted from TVP, MJ, and the public domain. Legal vs nonlegal ownership abstracted only from TVP.

c Information about the perpetrator(s)’ prejudice and motive(s) were abstracted directly from TVP. Mass shootings listed as “Unavailable” were not recorded in the TVP.

*CPMS*, civilian public mass shootings; *SAAR*, semi-automatic assault rifle; *SAP*, semi-automatic pistol.

**Table 2:** Mass Shooting Injury Matrix (classification of trauma by body region and nature of the injury) sustained by 364 patients injured in 13 civilian public mass shootings in the US (2012–19).^*^

Body region (33 Rows)		Nature of injury (12 Columns A-L)												Total diagnoses by body region

		A	B	C	D	E	F	G	H	I	J	K	L	
		Fracture	Dislocation	Sprain and strain	Internal organ trauma	Gunshot wound	Amputation	Vascular trauma	Soft tissue contusion	Crush Injury	Laceration and abrasion	Nerve injury	Other and unspecified	
Traumatic Brain Injury (TBI)														
1	Intracranial	5^a^	-	-	13^b^	-	-	-	-	-	-	0	-	18
2	Extracranial	3^a^	-	-	0	-	-	-	-	-	-	-	-	3
Head, Face & Neck														
3	Head and Scalp	0	-	-	-	13	-	0	5	0	3	0	13^c^	34
4	Face^d^	12	0	0	-	7	-	0	3	0	4	0	0	26
5	Eye and Eyelidd	-	-	-	-	8	-	0	3	0	0	1	0	12
6	Neck	0	-	4	-	4	-	1	0	0	1	0	0	10
7	Other and Unspecified	0	-	0	-	1^e^	-	0	0	0	0	0	2^f^	3
Spinal Cord Injury (SCI)														
8	Cervical SCI	0	-	-	0	-	-	-	-	-	-	-	-	0
9	Thoracic/Dorsal SCI	0	-	-	0	-	-	-	-	-	-	-	-	0
10	Lumbar SCI	0	-	-	0	-	-	-	-	-	-	-	-	0
11	Sacral/Coccygeal SCI	0	-	-	0	-	-	-	-	-	-	-	-	0
12	Unspecified SCI	0	-	-	0	-	-	-	-	-	-	-	-	0
Vertebral Column Injury (VCI)														
13	Cervical VCI	0	0	2	-	-	-	-	-	-	-	-	-	2
14	Thoracic/Dorsal VCI	1	0	1	-	-	-	-	-	-	-	-	-	2
15	Lumbar VCI	3	0	2	-	-	-	-	-	-	-	-	-	5
16	Sacral/Coccygeal VCI	0	0	0	-	-	-	-	-	-	-	-	-	0
17	Unspecified VCI	0	0	0	-	-	-	-	-	-	-	-	-	0
Torso														
18	Chest and Thorax	23^g^	0	2	45^h^	33	-	0	10	0	0	0	0	113
19	Abdomen	-	-	-	48^i^	33	-	2	4	1	1	0	0	89
20	Pelvis and urogenital	7	0	0	11^j^	9	-	0	0	1	0	0	0	28
21	Back and buttock	-	-	5	-	27	-	0	3	1	1	0	0	37
Upper extremity														
22	Shoulder, upper arm and axilla	17^k^	2	7	-	36	0	13	4	0	3	7	0	89
23	Forearm and elbow	14	2	1	-	22	0	3	5	0	9	4	0	60
24	Wrist, hand and fingers	30	0	8	-	23	2	1	4	0	8	3	0	79
25	Other and unspecified	0	0	0	-	15	0	0	1	0	1	0	0	17
Lower extremity														
26	Hip	8^l^	0	2	-	7	0	0	1	0	0	0	0	18
27	Upper leg and thigh	8^l^	0	1	-	39	0	3	1	0	3	4	0	59
28	Knee	4	0	1	-	7	0	2	5	0	4	0	0	23
29	Lower leg and ankle	18	1	7	-	32	0	5	3	0	6	1	0	73
30	Foot and toes	14	0	2	-	13	0	0	0	0	5	1	0	35
31	Other and unspecified	0	0	0	-	28	0	2	0	0	3	2	0	35
Multiple and unspecified														
32	Multiple body regions	1^m^	0	0	0	6^n^	0	0	3^o^	0	6^o^	0	0	16
33	Unspecified body region	6	0	0	0	0	0	2	0	0	2	1	0	11
Total diagnoses by nature of injury (Percentage of grand total diagnoses [N=897])		174 (19.4%)	5 (0.5%)	45 (5.0%)	117 (13.0%)	363 (40.5%)	2 (0.2%)	34 (3.8%)	55 (6.1%)	3 (0.3%)	60 (6.7%)	24 (2.7%)	15 (1.7%)	N=897
Diagnoses caused by GSW (Percentage of column total)		163 (93.7%)	0 (0.0%)	12^p^ (26.7%)	113^q^ (96.6%)	363 (100.0%)	2 (100.0%)	34 (100.0%)	0 (0.0%)	0 (0.0%)	0 (0.0%)	24 (100.0%)	14^r^ (93.3%)	725 (80.8%)
Diagnoses caused by non-GSW (Percentage of column total)		11 (6.3%)	5 (100.0%)	33^p^ (73.3%)	4^q^ (3.4%)	0 (0.0%)	0 (0.0%)	0 (0.0%)	55 (100.0%)	3 (100.0%)	60 (100.0%)	0 (0.0%)	1^r^ (6.7%)	172 (19.2%)

a Intracranial TBI, Fracture (1A) consists of 5 open skull fractures whereas Extracranial TBI, Fracture (2A) consists of 3 closed skull fractures.

b Intracranial TBI, Internal Organ Trauma (1D) consists of 6 cerebral lacerations/hemorrhages/infarctions, 4 subarachnoid hemorrhages, 2 subdural hemorrhages/hematomas, and 1 epidural hemorrhage/hematoma.

c Head and Scalp, Other & Unspecified (3L) consists of 13 closed head injuries/concussions caused by falls and other non-ballistic trauma.

d Face, Fracture (4A) includes orbital fractures but Eye and Eyelid (Row 5) includes all other trauma to the orbital region: 8 orbital GSWs (5E), 3 orbital contusions (5H), and 1 optic nerve/visual pathway injury (5K).

e Other Head, Face and Neck, Gunshot wound (7E) consists of 1 GSW to the ear.

f Other Head, Face and Neck, Other & Unspecified (7L) consists of 2 dental fractures (1 each by GSW and non-GSW).

g Chest and Thorax, Fracture (18A) includes open and closed rib fractures counted individually for each rib in the event of multiple fractures.

h Chest and Thorax, Internal Organ Trauma (18A) includes pulmonary contusion, hemothorax, pneumothorax, diaphragm injury, and myocardial infarction.

i Abdomen and Retroperitoneum, Internal Organ Trauma (19D) includes visceral trauma to the abdominal and retroperitoneal compartments including stomach, small intestine, large intestine, rectum, anus, liver, gallbladder, pancreas, spleen, and kidneys.

j Pelvis and Urogenital, Internal Organ Trauma (20D) includes visceral trauma to the lower urinary tract (ureter, bladder, and urethra), gonads (ovaries, testes), external genitalia, and uterus.

k Shoulder, Upper Arm and Axilla, Fracture (22A) includes all humerus fractures (proximal, mid-shaft, and distal), clavicle, and scapula fractures.

l Hip, Fracture (26A) consists of femoral neck fractures, including pertrochanteric fractures (greater and lesser trochanter). Upper Leg and Thigh, Fracture (27A) consists of all other femur fractures distal to the femoral neck including mid-shaft and condylar.

m Multiple Body Regions, Fracture (32A) consists of a single diagnosis code describing multiple metacarpal fractures but the total number was unknown. Therefore, one hand fracture (24A) and one multiple fractures (32A) were entered into the matrix.

n Multiple Body Regions, Gunshot Wound (32E) consists of 6 diagnosis codes describing GSWs of multiple sites of the arm or leg but the total number of GSWs was unknown. Therefore, one GSW to unspecified region of arm (25E) or leg (31E) and one multiple GSWs (32E) were entered into the matrix.

o Multiple Body Regions, Soft Tissue Contusion (32H) and Multiple Body Regions, Lacerations and Abrasions (32J) include 3 and 6 discrete diagnosis codes describing multiple injuries, respectively. Unlike multiple fractures and GSWs, multiple contusions and abrasions were only entered into the matrix once.

p Sprain, Strain and Tendon Injury (Column C) consists of 33 musculoskeletal strains and sprains caused by non-ballistic trauma and 12 tendon ruptures/injuries caused by GSWs.

q Internal Organ Trauma (Column D) includes 4 myocardial infarctions, 2 associated with blunt force trauma, and 2 associated with exertion/preexisting coronary disease. Otherwise, all other visceral trauma was caused by GSWs.

r Other and Unspecified (Column L) consists of 13 closed head injuries from falls/non-ballistic trauma, 1 dental fracture from a fall, and one 1 fracture from GSW.
